# Enhancing Robotic Perception through Synchronized Simulation and Physical Common-Sense Reasoning †

**DOI:** 10.3390/s24072249

**Published:** 2024-03-31

**Authors:** Guillermo Trinidad Barnech, Gonzalo Tejera, Juan Valle-Lisboa, Pedro Núñez, Pilar Bachiller, Pablo Bustos

**Affiliations:** 1 Facultad de Ingeniería, Universidad de la República, Montevideo 11200, Uruguay; 2 Facultad de Ciencias, Universidad de la República, Montevideo 11200, Uruguay; 3 Computer and Communication Technology Department, Universidad de Extremadura, 10005 Cáceres, Spain; pnuntru@unex.es (P.N.); pilarb@unex.es (P.B.);

**Keywords:** intuitive physics, cognitive robotics, manipulation

## Abstract

We introduce both conceptual and empirical findings arising from the amalgamation of a robotics cognitive architecture with an embedded physics simulator, aligning with the principles outlined in the intuitive physics literature. The employed robotic cognitive architecture, named CORTEX, leverages a highly efficient distributed working memory known as deep state representation. This working memory inherently encompasses a fundamental ontology, state persistency, geometric and logical relationships among elements, and tools for reading, updating, and reasoning about its contents. Our primary objective is to investigate the hypothesis that the integration of a physics simulator into the architecture streamlines the implementation of various functionalities that would otherwise necessitate extensive coding and debugging efforts. Furthermore, we categorize these enhanced functionalities into broad types based on the nature of the problems they address. These include addressing challenges related to occlusion, model-based perception, self-calibration, scene structural stability, and human activity interpretation. To demonstrate the outcomes of our experiments, we employ CoppeliaSim as the embedded simulator and both a Kinova Gen3 robotic arm and the Open-Manipulator-P as the real-world scenarios. Synchronization is maintained between the simulator and the stream of real events. Depending on the ongoing task, numerous queries are computed, and the results are projected into the working memory. Participating agents can then leverage this information to enhance overall performance.

## 1. Introduction

According to G. Hesslow (2002) [1], the concept of the *simulation hypothesis* posits that a simulated action can evoke perceptual activity resembling the activity that would have transpired had the action been physically executed. This notion is closely related to the field of *intuitive physics*, which has gained increasing attention in recent years. As articulated by Kubricht (2017) [2], “…humans are able to understand their physical environment and interact with objects and substances that undergo dynamic state changes, making at least approximate predictions about how observed events will unfold”.

Moreover, recent experiments provide compelling evidence that humans may possess an embedded mental game engine to aid in reasoning about their environment [3,4]. This research is intricately linked to the realm of robotics, with early antecedents in perception, as evidenced by experiments demonstrating internal visualization for navigation [5,6]. In architectural developments, the incorporation of an internal simulator into SOAR has been explored [7]. Despite objections raised by some authors who argue that the theory is only applicable when both time and space scales are small [8], ongoing advancements in simulation technology and computational power are continuously expanding these limits, as demonstrated by new applications [9,10,11].

In this paper, we explain in more depth the findings reported in our previous conference paper [12] related to incorporating a Physics Simulator into a Robotics Cognitive Architecture (RCA), explore its potential in addressing well-known challenges in robot perception, inspired by insights from the intuitive physics literature and present a novel experiment using our model in combination with an expectation violation approach. Our primary goal is to integrate a simulation-based reasoning pipeline into CORTEX. We have identified four specific use cases to illustrate how simulation can be leveraged to: (I) rectify object poses perceived by the robot based on a physically plausible model, (II) identify errors in pose perception and implement an automatic recalibration procedure, (III) analyze the persistence of objects beyond the robot’s line of sight, and (IV) detect and resolve disturbances during task execution. The subsequent sections give a more detailed explanation of the CORTEX architecture, the utilization of simulation as an internal tool for comprehending complex scenes, and an account of the experiments conducted to validate our application of this concept.

## 2. The CORTEX Architecture

The CORTEX cognitive architecture is a highly dynamic framework that has evolved continuously since its inception in 2016 [13,14]. In its present iteration, CORTEX is designed as a distributed architecture centered around a working memory (WM) and a set of agents with access to it. The working memory is formally represented as a directed graph where vertices hold metric or symbolic data, and edges signify geometric or logical predicates. The nodes or vertices represent concepts within the ontology, and the edges denote relationships between them. Functioning as a working memory, deep state representation (DSR) aims to depict the current situation involving the robot’s body, intentions, and the surrounding space and objects relevant to the ongoing task. The agents play a crucial role in creating and maintaining this representation, implementing functionalities such as perception and motor modules, along with procedural, declarative, and episodic memories in alignment with the standard model [15].

The WM in CORTEX is implemented as a very efficient distributed graph data structure using a three-layered design: a DDS middleware implementation that provides reliable multicast, a CRDT graph implementation that provides eventual consistency, and a simple high-level API to edit elements of the graph.

CORTEX operates within the working memory by establishing a specific structure. The node representing the robot maintains a constant connection through an *RT* (rotation–translation geometric transformation) edge to one of the existing nodes that signifies a spatial zone, typically a room in indoor settings. This edge undergoes modification whenever the robot changes its location. Stemming from the robot, two distinct nodes, namely, *body* and *mind*, are linked downward. The remaining components of the robot are connected to the *body* node based on their kinematic relationships. These components encompass rigid segments, joints, sensors, and actuators. The raw data generated by sensors are stored in the node’s attributes, ensuring accessibility to all agents. This streamlined data sharing is facilitated by an efficient software design and implementation [16,17].

The other branch, *mind*, encapsulates the current intention or goal of the robot. This intention is translated into plans by deliberative agents, generating additional nodes branching from *intention* to store and broadcast the current list of subgoals or actions. All agents are informed about the prevailing intention and plans, enabling them to take actions aligned with their functionalities to achieve the goal. Once the goal is accomplished, the intention node is removed, and the agents revert to their respective local activities.

**Figure 1:**
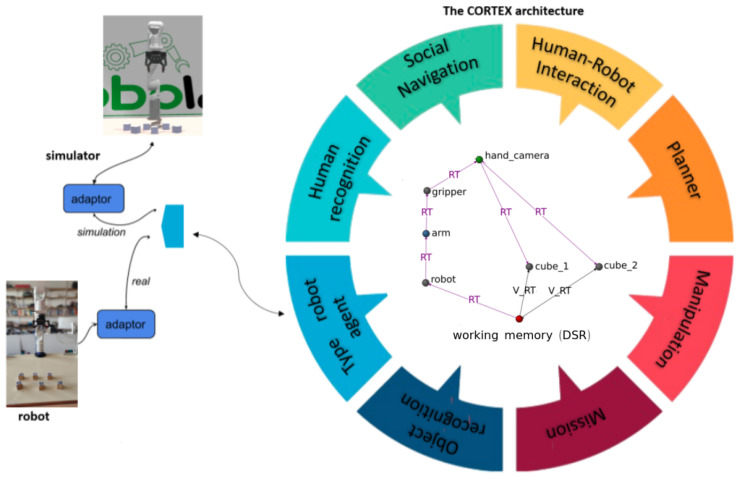
The figure shows an instance of the CORTEX architecture with a double connection to the robot’s body and a simulator. The working memory is instantiated as a directed graph (see details of the graph in Figure 2).

**Figure 2:**
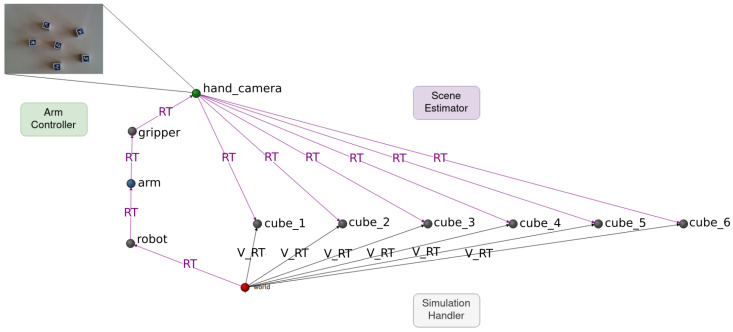
Example DSR state. Arm controller writes camera readings as an attribute of the hand_camera node (top). Position estimations of the cubes (middle) are presented as RT edges from *hand_camera*, inserted by the scene-estimator agent. Virtual_RT edges (named V_RT in this figure) represent geometric transformations from the origin (*world* node, bottom) to every cube and are given by the simulation-handler agent.

Intentions are overseen by a specialized agent known as the *mission manager*, which typically provides a graphical user interface (GUI) to the roboticist, accepts new missions from interacting humans, or utilizes a scheduler to activate periodic missions. Figure 1 presents an abstract depiction of a CORTEX deployment, while Figure 2 illustrates a real-world scenario of object detection and localization. For a more in-depth understanding of the CORTEX implementation, additional details can be found in [18].

## 3. The Benefits of Embedded Simulation

The working memory in CORTEX furnishes a lasting, organized state representation, facilitating the implementation of intricate cognitive-mediated behaviors. However, in certain situations, relying solely on direct perception and persistence may not suffice to address straightforward problems. What may be essential is a kind of pre-existing knowledge that can be injected into the working memory to offer a foundational form of common sense. This knowledge can stem from various sources and manifest in diverse formal representations. In cognitive robotics, a typical source is an ontology with robust relationships among concepts and an inference engine for responding to queries, as demonstrated in [19].

In our work, we aimed to acquire predicates that establish relationships among objects in the current scene, with a specific focus on predicates derived from a physics simulator synchronized with the working memory. To systematically explore this approach, we examined examples and scenarios where incorporating this common-sense knowledge would notably enhance task resolution. These examples were categorized informally, emphasizing common patterns. Here, we discuss four of them, as mentioned in the Introduction, while the remaining scenarios will be briefly addressed in the Conclusions section as ongoing research directions.

## 4. Experimental Setup

The experimental setup comprised a Kinova Gen3 (Boisbriand, QC, Canada) (or Open Manipulator Pro developed by Robotis (Chiyoda, Tokyo)) robotic arm positioned on a table and a set of 4 cm × 4 cm cubes, each marked with a distinct AprilTag [20] on one face. Attached to the wrist of the robotic arm was an RGBD camera (Realsense D415 (Intel RealSense, Santa Clara, CA, USA)). The simulation was conducted using CoppeliaSim V4.4 (Zurich, Switzerland)  [21].

**Figure 3:**
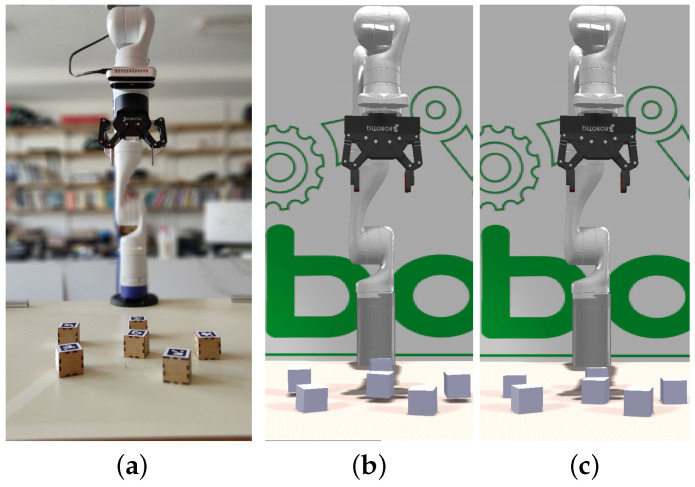
Upon encountering state (**a**), the robot utilizes RGBD information to derive estimation (**b**), which proposes poses where cubes seem to hover above the table. Following the application of simulation physics, scene (**c**) is produced, rectifying the initial misperceptions.

To initialize the working memory in CORTEX, a file is used containing a subgraph that represents the robot and its sensors, along with additional nodes representing the scenario. An illustrative configuration is depicted in Figure 3, where (a) showcases the real-world state, and (c) demonstrates its reproduction within CoppeliaSim. All simulations were executed at a rate of 20 Hz on an Intel i9 10th generation processor (Santa Clara, CA, USA) and an RTX3090 GPU (NVIDA, Santa Clara, CA, USA).

For these experiments, three CORTEX agents were developed and deployed, each with specific roles:

1. **Arm controller**: This agent operates in real time, reading and inserting the gripper pose from the Kinova arm into the working memory as an SE(3) spatial transformation originating from the arm base. Additionally, it injects the raw RGBD data stream acquired from the RealSense camera as an attribute of the *hand_camera* node.
2. **Scene estimator**: Responsible for detecting AprilTags, this agent inserts model cubes into the working memory. The cubes are linked to the *camera* node through an RT edge, incorporating the estimated relative pose.
3. **Simulation handler**: Tasked with bidirectional synchronization between the working memory and the simulation, this agent reads cube poses from the graph to update the simulation and publishes them back as *Virtual_RT* edges. These *Virtual_RT* edges, while not part of the RT tree to avoid inducing loops, are treated as symbolic edges, representing an *opinion* from the simulator.

In CORTEX, agents operate autonomously and communicate indirectly through the working memory. An illustrative state of the deep state representation is presented in Figure 2, encompassing all the information contributed by the agents outlined earlier.

We now describe three experiments, in which the use of the same integrated simulator facilitates the approach to three well-known problems in robotics.

### 4.1. Model-Based Perception

The robot’s perception of objects in the world is inherently prone to noise and positioning errors. In scenarios where objects are positioned atop or leaning over others, these perception errors can lead to physically implausible configurations, such as floating or intersecting objects. To address this, the simulator can leverage internal physics laws to swiftly identify a feasible configuration, correcting for the errors and projecting the corrected configuration back into the working memory. Consequently, the robot perceives a configuration of objects that has been refined by a sophisticated internal model adhering to the principles of physics (Figure 3). By embedding this pipeline within the working memory, all agents gain access to this form of physical reasoning, enhancing their comprehension of the observed scene.

This functionality has been exemplified in previous works, such as [11,22]. In the subsequent sections, we elaborate on how this capability can enhance the robot’s overall performance and precision.

### 4.2. Self Calibration

Due to model-based perception, a residual error is computed between the estimated pose and the model-corrected pose. This error, often systematic, can stem from an incomplete calibration of the robot’s sensors. The primary cause is typically a misalignment of the sensing device in the kinematic chain, such as an RGBD camera or LIDAR, often hard-coded by a human. In such cases, the computed error can be employed to recalibrate the sensor by calculating the corresponding derivatives.

Let $c_{i}^{r}$ represent the real-world pose of cube *i*, $p^{r}$ denote the camera’s pose relative to its parent frame in the kinematic chain, and $c_{i}^{e}$ signify the pose estimated by the system for cube *i*. Utilizing $C^{e}$ (the set of all $c_{i}^{e}$), the simulation was synchronized, and after applying physics, $C^{s}$ was obtained (${⋃}_{i}c_{i}^{s}$ where $c_{i}^{s}$ is the corrected pose for cube *i*). In the following experiment, our objective was to determine a value for $p^{r}$ that minimized the distance between $C^{e}$ and $C^{s}$, using the simulator’s corrections as an approximation for $C^{r}$. The error function utilized was the average distance across all detected cubes, as described in Equation (1), and the Powell minimization method from Scipy was employed for the implementation.

(1) $$dist\left(c_{i},c_{j}\right)={\langle c_{i}^{rot},c_{j}^{rot}\rangle }^{2}+\left|\right|c_{i}^{trans}-c_{j}^{trans}\left|\right|$$

Recalibration may be necessary due to two distinct scenarios: (i) a well-calibrated system undergoes some change, such as unexpected sensor movement, or (ii) the initial sensor position is erroneous, requiring the determination of the correct one. In this discussion, we focus on the first scenario, but the same process can be applied for the second.

Each time a new cube was detected, it was incorporated into the simulation. In this situation, the robot rediscovered previously placed cubes, yet the disparity between $C^{e}$ and $C^{s}$ (computed with the error function) was substantial. While various events could account for this difference, a recalibration was initiated to assess whether a modification in the camera pose could rectify the error. If a value for $p^{r}$ aligning $C^{e}$ to $C^{s}$ was identified, it was adopted as the new camera position going forward.

**Figure 4:**
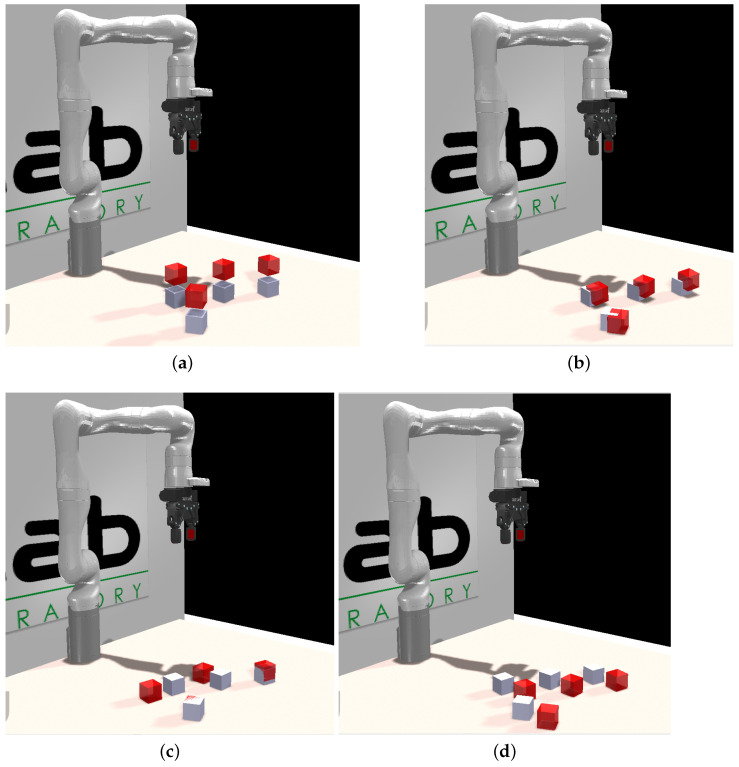
Possible scenarios for recalibration. In red are shown the cube positions reported by the perceptual system($C^{e}$) and in white those previously inserted in the simulator ($C^{s}$). (**a**,**b**) represent cases of systematic error, where a correction in the camera position explains the discrepancy. (**c**) shows an unsystematic error, where some cubes moved, but not coordinately and (**d**) presents the case where the cubes were moved coordinately, indistinguishable for the system from cases (**a**,**b**).

However, it is crucial to note that this improvement could be a result of a coordinated movement of all the cubes, creating the appearance of sensor displacement. Although this scenario is considered less likely than a calibration error, if it were the case, the robot would detect a systematic error in its perceptions and initiate recalibration once again. On the contrary, if the error prompting the recalibration process stems from one or more cubes being moved, a modification in the camera position would not align these new positions with those of the simulator. Consequently, the minimization would fail, and the previous configuration would be retained. The three cases are illustrated in Figure 4, where both Figure 4a,b represent scenarios where a systematic error is identified, correctable by adjusting the camera slightly towards the table or rotating it on its axis, respectively. In Figure 4c, we observe a case where some cubes remained in place while others showed displacement. This form of nonsystematic error could not be rectified by recalibration, and it was interpreted as the cubes having simply changed their position. The last image (Figure 4d) depicts a scenario where all cubes were moved uniformly in the same direction and distance. In this situation, the system would erroneously recalibrate the camera position, but subsequent encounters with objects should trigger a second calibration stage to rectify this.

To assess this functionality, a straightforward scene was constructed, akin to the one depicted in Figure 3. Once the cubes were detected and integrated into the simulation, the last transformation in the kinematic chain to the camera ($p^{r}$) was intentionally distorted. Specifically, the spatial transformation from the arm’s tip to the camera was replaced with a random one. This manipulation achieved the desired effect, where all cubes remained visible, but the disparity between detected ($C^{e}$) and remembered ($C^{s}$) positions was larger than expected.

**Figure 5:**
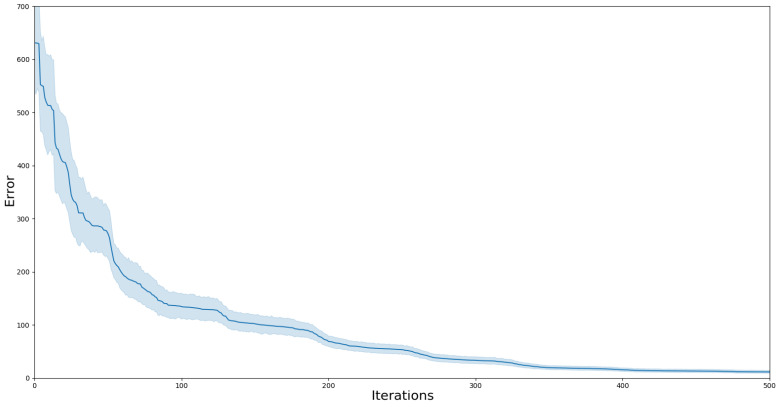
Results of the recalibration after an undesired change in camera position. The blue line represents the mean across trials with the 95% confidence interval, showing how the system can find a low-error solution despite initiating from highly erroneous guesses.

**Figure 6:**
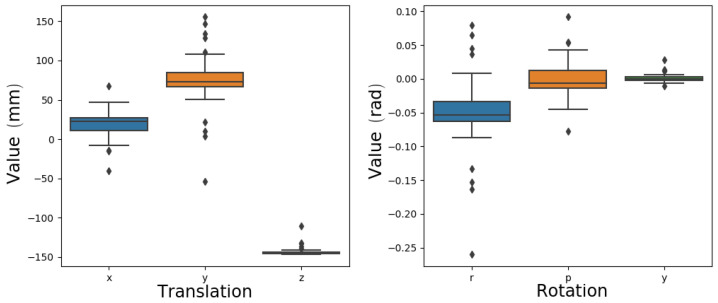
Values for translation (x, y, z) and rotation (raw, pitch, yaw) from the arm tip to the camera found across trials.

Using a setup with four cubes and a top view, 50 trials of this experiment were executed. Figure 5 illustrates the evolution of the error during the optimization process. This graph demonstrates that the method can attain low error values for $p^{r}$ even when commencing from highly erroneous initial guesses. While convergence does not guarantee that $p^{r}$ is the actual pose, the results indicate a low variance across trials, and the estimated position of the camera is close to the measured position (Figure 6).

#### Limitations

Following the results obtained in the first tests, it was decided to study the possible limitations for this type of process. In particular, we sought to investigate whether there was a minimum number of objects for this to work and whether the positions of these objects in the work area affected the quality of the calibration.

The procedure for testing the different variables when calibrating was similar in both tests. First, the system was initialized with a value of $p^{r}$ with a low precision, in particular, a precision of one centimeter was used when measuring each of its translation values. The system placed the detected cubes in the simulator and waited for it to correct them. The system was calibrated, and the values of the proposed transformation were saved.

**Figure 7:**
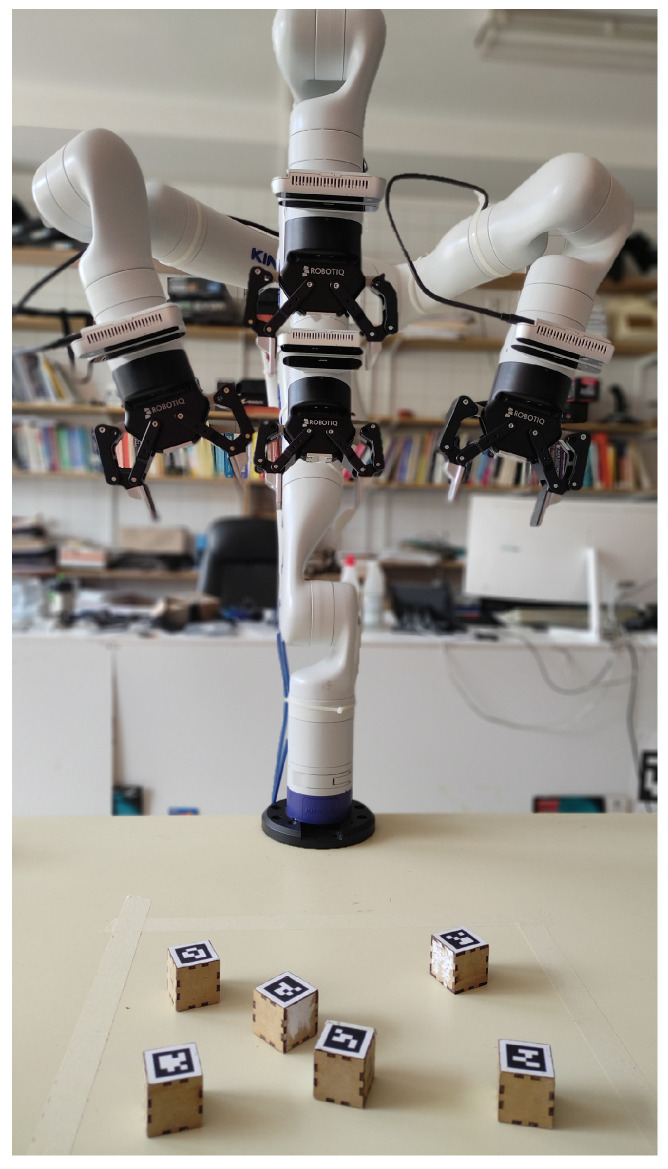
Overlapped arm positions used to evaluate the consistency in the estimates generated by the proposed camera positions in the calibration phase.

To evaluate each transformation, we initialized the system with its value and waited for it to place the cubes in the simulation. Then, the cubes were observed from four different arm positions, measuring the error between the estimation of the cubes’ positions from that point of view and their position in the simulation. Using multiple viewpoints evaluated the consistency of the transformation, since if we were to use only one, it may be conforming to that perspective. Figure 7 shows an overlay of the postures used in the evaluation. Six cubes were used for this evaluation, but some were discarded due to a very high level of error in all cases. This error can be explained by problems in the detection of their AprilTag, either because of stains, tag malformations, or the illumination at the time.

**Number of cubes in the scene**

**Figure 8:**
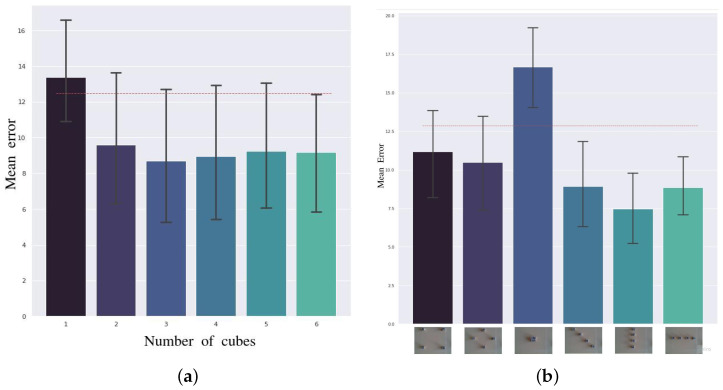
Self-calibration robustness. (**a**) Average error according to the number of cubes used for calibration. (**b**) Mean error according to the cube configuration used for calibration. The dotted line in both figures represents the error using the transformation before calibration.

For this experiment, the system was calibrated by varying the number of cubes. For each quantity, three calibration sessions were performed. The combined results of these are presented in Figure 8a. The dashed red line represents the error made by the system using the initial, i.e., precalibration, transformation.

As expected, using a single cube did not improve the performance, since the transformation was adjusted so that the error was zero at calibration, overfitting to that point of view. From two cubes on, the system started to have errors below those made before calibration and from three, the confidence interval remained almost completely below the baseline. The error differences between the latter cases were small and nonsystematic.

**Cube configuration**

In the previous test, the cubes’ positions when calibrating the system were chosen randomly using a simple script. But we also investigated how these might affect the quality of the calibration. For this purpose, six symmetric configurations were designed with the intention of exploring whether any distribution was particularly beneficial.

As can be seen in Figure 8b, whenever the cubes were found to cover the entire working area, the calibration improved with respect to the initial one. The configurations that made the least error were those that covered it most densely. When the cubes were close together, as in the third case, the calibration resulted in a worse performance. This result did not deviate from what was expected. When the cubes were so close together that the situation resembled that of having only one cube, the system could eliminate the supposed systematic error by adjusting to this particular situation but generating an incorrect transformation.

### 4.3. Occlusion

When the robot loses sight of an object that is being tracked, its presence and continuity in the working memory (WM) need to be updated through internal reasoning processes. As an illustrative example of occlusion, a recent experiment [11] demonstrates a human holding a ball over two boxes and subsequently dropping it inside one of them. The boxes are then interchanged, and the system is queried about the ball’s location. For a robotics cognitive architecture with an embedded simulator, answering this query is relatively straightforward by allowing gravity and collision detection algorithms to operate on the free ball.

Once the system introduces any object into the simulator, its position is consistently recorded in the WM as a virtual RT. Unseen interactions are automatically calculated, and the position information is readily available at any time to any agent requiring it.

In contrast to the approach taken by Sallami et al. [11], where human intervention was detected through a state machine observing object configurations strongly violating the effect of gravity, we leveraged the geometric reasoning within our setup to detect grasping. A novel agent was developed, employing the MediaPipe Hands algorithm [23] along with depth information to detect human hands. This agent then placed the finger positions into the working memory. Subsequently, the *simulation-handler* agent incorporated these fingertips into the simulation as simple spheres. As long as one or more of these spheres were in collision with an object, gravity was not applied to it.

Although the algorithm does not explicitly differentiate between grasping and touching, this example illustrates how having a physical duplicate of reality can equip the system with straightforward means of detecting complex interactions, such as contact and gravity. These interactions would be challenging to describe using rules or ad hoc algorithms alone.

**Figure 9:**
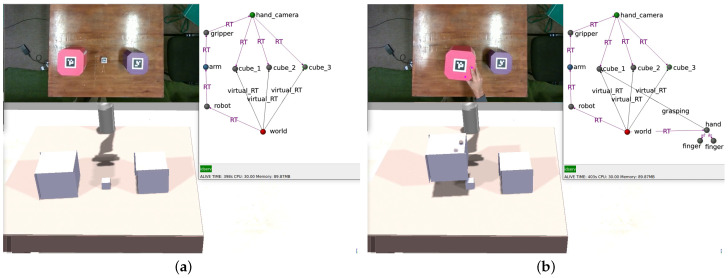

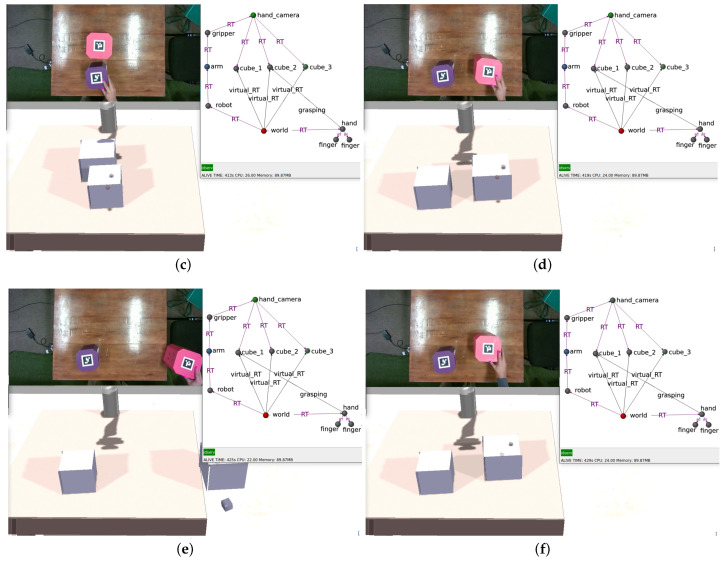
Cups and balls experiment. (**a**) Shows the initial configuration. When a human hand is detected for the first time, a new node is inserted, and two spheres representing fingertips placed in the simulation (**b**). Grasp detection can be seen in (**c**,**d**) where the WM has a *grasping* edge from the hand to the box being manipulated. In (**e**), the box containing the cube is placed over the edge, and the simulator shows the cube falling to the ground. (**f**) shows the final state of the experiment.

Employing these elements, we conducted a similar experiment to replicate the classic game of cups and balls (Figure 9). The robot was presented with a cube and two boxes (Figure 9a, the boxes’ shapes were approximated with hollow prisms for simplicity). One box was positioned over the cube (Figure 9b), and then the boxes were swapped (Figure 9d). At a certain point, the box containing the cube was moved over the edge of the table, causing it to drop to the floor (Figure 9e). Thanks to the synchronized simulator, the system could detect this situation and continued to accurately track the cube’s position, even though it was out of sight for most of the time. This ability to determine the cube’s location without relying on extensive sets of rules or case-specific heuristics exemplifies the simplicity and advantages of our approach. Through internal simulation, this information remains readily accessible to the robot, while using rules requires the system to explicitly represent the interactions. For example, one could argue that for this specific scenario two rules suffice: an object inside a container will move with it, and an object will fall if not supported by a surface or a grasping hand. However, several issues arise when grounding these rules: What does it mean for an object to be inside another object? In what way will it move with it? How can you determine if an unobserved object is being supported by a surface? And above all, these two rules contradict each other when the container is supported, but the contained object is not: should the latter move with its container or fall because it is not supported? All these issues are implicitly solved by our approach, where the interactions naturally occur inside the simulation.

### 4.4. Expectation Violation during Plan Execution

During tests with the system, some particular situations were identified in which the simulator could not explain the perception. At times the perception detected objects in positions far away from those found in the simulator. This raised the question: who should I believe? And the answer is not always the same. As an external observer, errors in perception can be identified with the same frequency with which synchronization errors are found. In many cases, these errors eventually converge to a stable and correct state, but the intermediate effects on unperceived objects may not be reversible.

One of the most common cases occurs when one cube is under another cube forming a tower, and the tower moves together to another location. When this happens, the system perceives the cube above but is constantly in a loop where the simulator makes the cube fall four centimeters and the perception detects it again in the air. This discrepancy could be detected as surprising and solved using semantic information about the state of the world, such as an edge *On*, indicating that previously, the floating cube was on top of another one.

#### 4.4.1. Surprise Detection Agent

These cases where perception does not match the mental model have been extensively studied in cognition. In the work of Smith et al. [24] this is called a violation of expectation, meaning that a particular observed situation does not match our expectation according to intuitive physics. This signal of surprise may be a way to detect errors, but for the same reasons, it is an opportunity to try to correct our mental model.

Inspired by this idea, we implemented an agent that was in charge of monitoring the state of the working memory and if necessary, triggering a surprise signal. In particular, this agent calculated at each moment the difference between the position of an object according to the perception (following the kinematic chain to it) and the mental model (represented by the Virtual_RT edges). If this difference exceeded a threshold, the agent modified the surprising attribute within that cube, indicating that it was in violation of the system’s expectations.

#### 4.4.2. The Task

In order to test the new surprise detection agent, the robot started with four cubes on the table and was given the task of creating two towers with two cubes each, but the state of the cubes was constantly perturbed by the experimenter. During this execution the hand recognition agent was turned off, so the cubes seemed to be moved by undetected events. A planning agent was developed, which used the available nodes and edges in the working memory to express the world state in PDDL [25] and the FastDownward [26] solver to find a plan.

During plan execution, the same agent inserted the effects of each action as edges in the working memory (i.e., after grasping, a grasping edge was inserted between the cube and the robot arm). When a change in the working memory state was detected and it was not a consequence of this agent, the execution halted, and the agent built the new initial state and replanned.

This unexpected changes in the working memory came from the effects of an expectation violation event. Once a cube was marked as surprising by the agent, the simulation handler agent had to return the system to a stable state, using semantic information to test some explanations. This process is described in Algorithm 1 and the ideas behind it are that if a cube is surprising and, according to the working memory, it should have another one underneath it, we try to explain this situation by placing them once again in that way. In the opposite case, if the robot perceives a cube that is supposed to be under another one, it means that this is no longer true, since it would be impossible to see it. Lastly, if the system has no semantic information, it simply updates the position inside the simulation with the new perceived pose.
**Algorithm 1** Handling of surprising cubes**Input:** $S\leftarrow \{c_{i}|c_{i}\in C,c_{i}.surprising=True\}$    **for** c∈S **do**         **if** $\exists c_{2}:on\left(c_{2},c\right)$ **then**              $c_{2}.position\leftarrow$ under c              simlation.update_position($c_{2}$)         **else if** $\exists c_{2}:on\left(c,c_{2}\right)$ **then**              dsr.delete ($on(c,c_{2})$)              simlation.update_position(*c*)         **else**              simlation.update_position(*c*)         **end if**    **end for**

**Figure 10:**
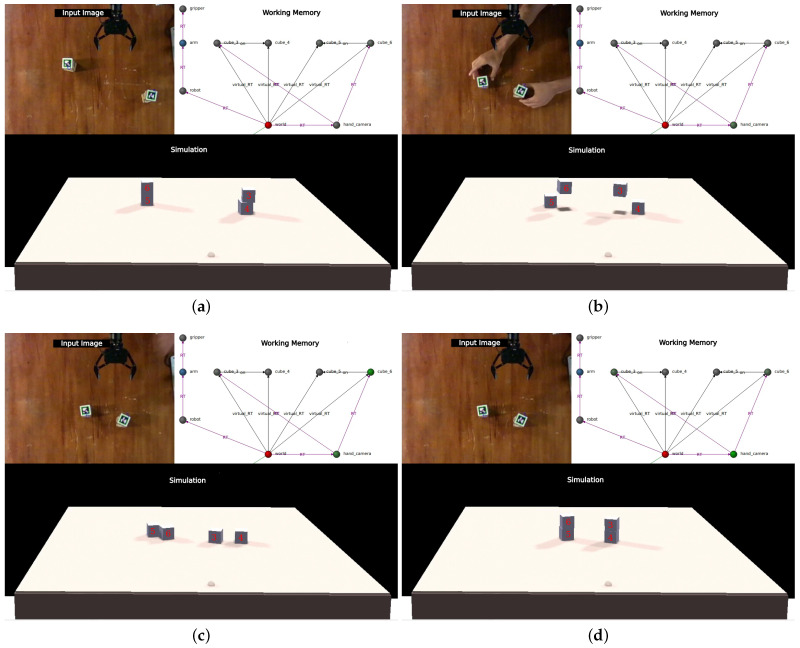
Full-tower movement. The system maintains the final state well represented in (**a**) until the experimenter moves both towers out of place (**b**). These new positions trigger a surprise signal, since there is no match between perception and simulation (**c**). By consulting the working memory, the system tries to explain this situation by placing cubes four and five below cubes three and six, respectively (**d**). This modification results in a stable state, so it is taken as the explanation of the situation.

**Figure 11:**
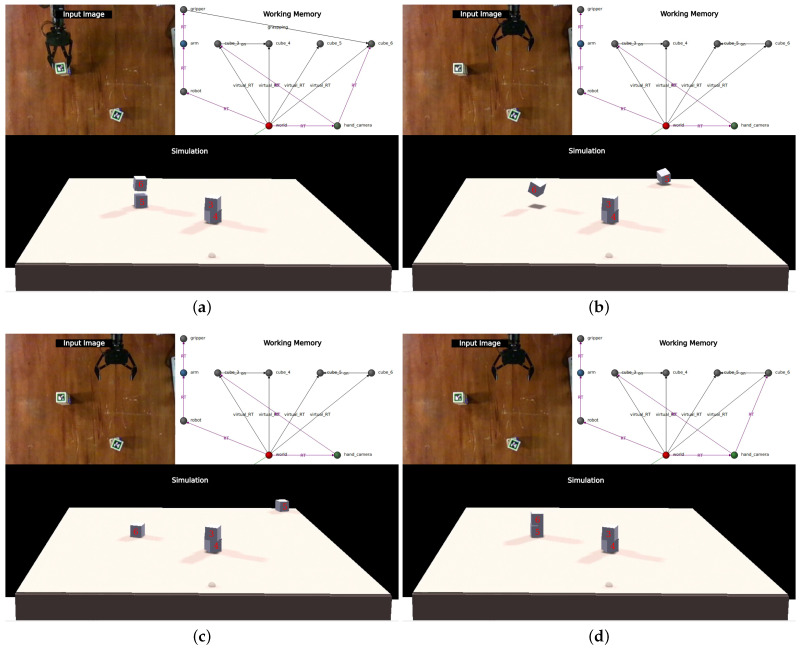
Correction of errors in the simulation. While the arm was placing one cube on top of the other (**a**), an error in positioning causes the one below to shoot out (**b**). When this happens and the cube is no longer being grabbed, it can be seen in the simulation that it falls on the table, but this position does not match the perception and triggers a surprise signal (**c**). Again, thanks to semantic information, the cube is placed under the other and a stable configuration is achieved (**d**).

**Figure 12:**
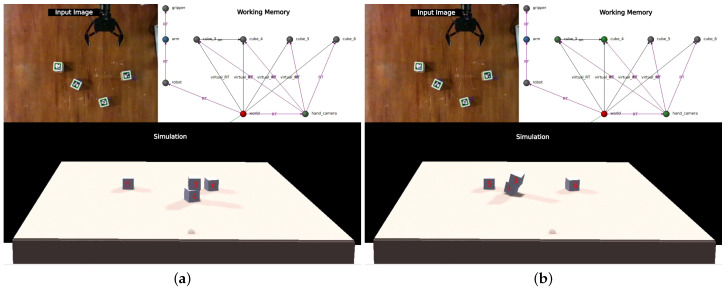

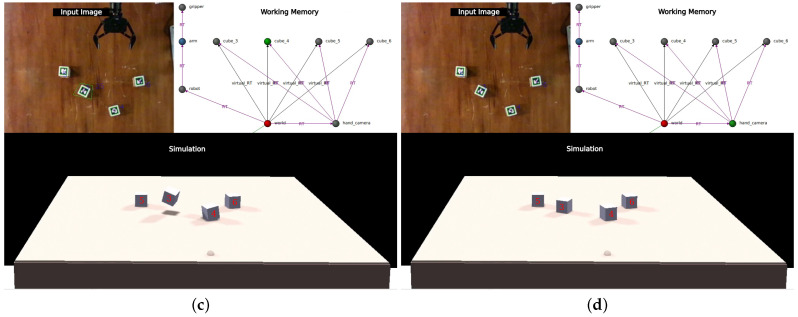
Tower disassembly. The robot has already formed the first tower, placing cube three on top of cube four. But before it can go for the second, the experimenter places cube three back on the table (**a**). Perceiving cube three in this new location triggers the surprise signal, and since this cube was on top of four, the system places it underneath to test if that solves the discrepancy (**b**). As can be seen in (**c**), it is now cube four that generates the surprise, since by placing it underneath three its position disagrees with the perceived one. The system, as a reaction to this, erases the edge that connects the two cubes, managing to return to a harmonious state between the perception and the model (**d**).

**Figure 13:**
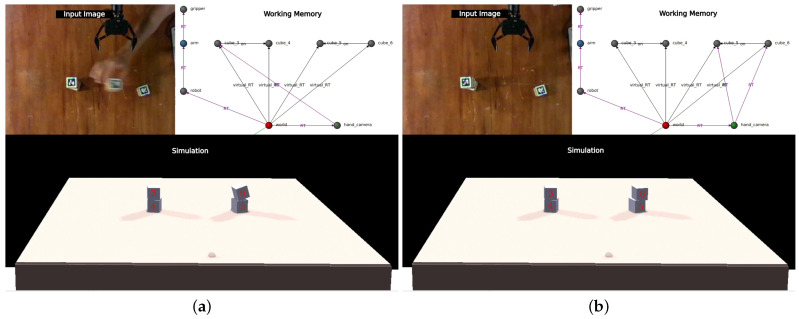

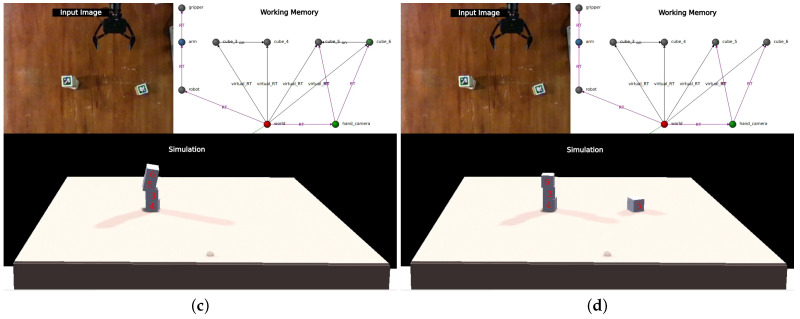
Three-cube tower. The robot has already finished assembling the two towers (**a**), and the experimenter places cube six on top of cube three (**b**). Upon detecting this cube out of the expected position and knowing its relation to cube five, an attempt is made to explain the new position by placing five underneath (**c**). Similarly to what happens in the previous test, the presence of cube five there disagrees with the perception and, therefore, it is positioned where it is perceived, and the edge that connects it with six is deleted (**d**). The state in which the simulation is left corresponds perfectly to reality, but the working memory does not fully capture the relationships between the cubes. With this state, the robot can take any of the cubes since the positions it contains for them are correct, but it will not be able to correct the simulation for some particular perturbations (such as moving the whole tower of three as a whole).

This simple process has proven to be sufficient to keep the simulation state synchronized during the following perturbations (videos documenting each of the tests are available in the *Maestría* section of https://www.fing.edu.uy/~gtrinidad, accessed 1 January 2024):

- **Full-tower movement**: It consists of moving a tower of two cubes and evaluating whether the system is able to recognize this perturbation and use the semantic information of the graph to correct it. Figure 10 shows snapshots of the execution of this perturbation.
- **Correction of errors in the simulation**: When a grasped cube collides with another one that is not being perceived at that moment, as the grasped cube does not respond to physics, when it collides with another one, it can apply a great force to it (since it is the second one that absorbs all the reaction). When this second cube is not being perceived, the system cannot return it to its place and the model remains in an inconsistent state. An example of this situation is presented in the Figure 11.
- **Tower disassembly**: The experimenter disassembles a tower in the middle of the plan, causing the robot to react, resynchronize, and re-plan. Figure 12 presents the execution of the test.
- **Three-cube tower**: As a final test and as a way to explore the reaction of the system to situations outside of its planning, this simple experiment is designed when the robot has already finished assembling the two towers (Figure 13a) and the experimenter places the cube from the top of one tower on top of the other.

For these experiments, the Open Manipulator Pro developed by Robotis (Chiyoda, Tokyo) was used, equipped with the RH-P12-RN gripper. Perception came from a RealSense D455 camera (Intel RealSense, Santa Clara, CA, USA) placed on a frame above the working area. It achieved real-time execution on a PC equipped with an NVIDIA 1650 graphics card, an Intel I7 (10th generation) processor, and 16 Gb of RAM.

## 5. Conclusions and Future Work

In this paper, we showcased several experiments conducted with a robotics cognitive architecture integrated with a physics simulator. The objective was to explore and test various applications of the newfound simulation capability, each enhancing desirable aspects of the architecture. Across the four examples, the fundamental mechanism remained consistent—the embedded simulator accessed the WM to write there the results of its own synthetic perception process—extending the original bottom-up perception approach with a top-down model-based complementary view. The coexistence of these to views provided an error measure for each represented object. How this error is used is an actively debated question in cognitive science, with K. Friston’s active inference theory [27] rapidly gaining momentum on the battlefield [28].

Aligning with the distributed and reactive nature of CORTEX, the injection of virtual RT edges by the simulator occurs asynchronously into the working memory. In contrast to necessitating explicit cues for accessing geometric knowledge, our system is propelled by activity within the working memory and responds by furnishing alternative RT edges. This approach will be further explored in the future, especially as additional common-sense agents managing declarative knowledge or episodic memories are developed.

In future work, we plan to enhance the simulation capabilities by incorporating various simplifying shortcuts to make real-time simulation feasible for a larger number of objects [29]. This is crucial for handling more realistic scenarios. Additionally, the simplifications will enable the deployment of multiple simulations and the implementation of a probabilistic learning approach that could facilitate the unsupervised training of the robotic arm in cluttered situations. Consequently, it will streamline the integration of probabilistic and neural network learning—a successful approach applied in other domains, such as program induction [30]. A first possibility is to extend the RT geometric links with covariance information obtained from the error model of the sensor and the measurement process. This would make them stochastic transformations that could be interpreted in the internal simulator as different initial conditions to be set before applying its internal dynamics. The result would be a set of possible outcomes, anticipating different courses of reality, entering the working memory to be pruned as new data arrive. A second possibility is to make the simulations stochastic by introducing uncertainty in the actions. This would also produce a set of possible outcomes covering the modeled uncertainties in the real actuators. Another interesting approach we are currently investigating is the use of a local large language model (LLM) as part of the cognitive architecture. It will play a different but complementary role to the internal simulator. We are exploring ways of verbalizing the state of working memory in a prompt and then asking for solutions to planning problems and for natural language explanations that can be offered to humans.

An additional promising avenue of research focuses on interpreting human activities [31]. One possibility could be implemented by combining the retargeting of the perceived body into the robot’s geometry and the retrieval of previous sensorimotor episodes in similar situations, such as grasping a box. Utilizing a synchronized simulation where the human hand is retargeted into the robot’s gripper, the physics engine can replicate the most similar gripper configuration for holding the object. Also, using the expectation violation paradigm, the robot could detect situations where the world has changed in a way only a human (or another agent) could have generated. This could be a cue for the robot to try and interpret what this agent was trying to do and, for example, change its plan to help him/her [32].

## Data Availability

The data presented in this study are available on request from the corresponding author.
